# RISK AND RESILIENCY IN THE RELATIONSHIP BETWEEN WIDOWHOOD AND DEPRESSIVE SYMPTOMS AMONG OLDER MEXICAN AMERICANS

**DOI:** 10.1093/geroni/igz038.3014

**Published:** 2019-11-08

**Authors:** Zhen Cong, Yaolin Pei, Bei Wu

**Affiliations:** 1 School of Social Work, University of Texas at Arlington, Arlington, Texas, United States; 2 NYU Rory Meyers College of Nursing, New York, New York, United States

## Abstract

This study investigated the association between widowhood and depressive symptoms and the extent to which the association is contingent upon immigration status, functional limitations, financial strains, and intergenerational support, among older Mexican Americans. A sample of 344 parent-child dyads reported by 83 older adults in a city of West Taxes completed the measures for socioeconomic status and depressive symptoms. Clustered regression analysis showed that widowhood elevated the risk of depressive symptoms. Living with functional limitations, having more children and residing in the same city with children exacerbated the adverse effects of widowhood on depressive symptoms. Residing in the same city with children increased the detrimental effects of widowhood on the depressive symptoms among men, whereas this pattern did not appear among women. The findings highlight the heterogeneity of depressive symptoms among the widowed Mexican American older adults.

